# Cross-cultural adaptation, validity and reliability of the Persian translation of the Western Ontario Shoulder Instability Index (WOSI)

**DOI:** 10.1186/s13018-023-03593-z

**Published:** 2023-03-07

**Authors:** Ehsan Kheradmand, Seyed Mohsen Rahimi, Morteza Nakhaei Amroodi, Parisa Nejati, Sharon Griffin

**Affiliations:** 1 Department of Sports and Exercise Medicine, Hazrat-e Rasool General Hospital, School of Medicine, Iran University of Medical Sciences, Tehran, Iran; 2 Department of Orthopedic Surgery, Bone and Joint Reconstruction Research Center, School of Medicine, Iran University of Medical Sciences, Tehran, Iran; 3 Fowler Kennedy Sports Medicine Clinic, University of Western Ontario, London, ON Canada

**Keywords:** Shoulder, Quality of life, Translation, Outcome measures, WOSI

## Abstract

**Purpose:**

The Western Ontario Shoulder Instability Index (WOSI) is the most commonly used patient-reported outcome measure to record the quality of life in patients with shoulder instability. The current study aimed to translate the WOSI into the Persian language and evaluate its psychometric properties.

**Methods:**

The translation procedure of the WOSI was performed according to a standard guideline. A total of 52 patients were included in the study and responded to the Persian WOSI, Oxford shoulder score (OSS), Oxford shoulder instability score (OSIS), and disabilities of arm, shoulder and hand (DASH). A sub-group of 41 patients responded for the second time to the Persian WOSI after an interval of 1–2 weeks. The internal consistency, test–retest reliability using intraclass correlation coefficient (ICC), measurement error, minimal detectable change (MDC), and floor and ceiling effect were analyzed. The hypothesis testing method was used to assess construct validity by calculating Pearson correlation coefficient between WOSI and DASH, OSS, and OSIS.

**Results:**

Cronbach's alpha value was 0.93, showing strong internal consistency. Test–retest reliability was good to excellent (ICC = 0.90). There was no floor and ceiling effect. The standard error of measurement and MDC were 8.30% and 23.03%, respectively. Regarding construct validity, 83.3% of the results agreed with hypotheses. High correlations were observed between WOSI and DASH, OSS and OSIS (0.746, 0.759 and 0.643, respectively) indicating excellent validity for the Persian WOSI.

**Conclusion:**

The current study results demonstrated that the Persian WOSI is a valid and reliable instrument and can be used in the clinic and research for Persian-speaking patients with shoulder instability.

**Supplementary Information:**

The online version contains supplementary material available at 10.1186/s13018-023-03593-z.

## Introduction

Shoulder instability includes discomfort and diminished shoulder joint function due to abnormal movement of the humeral head in the glenoid cavity [1]. This disorder may be reported by the patient as pain, a feeling of abnormal shoulder movement, or a feeling of shoulder dislocation or subluxation [2, 3]. When this happens repeatedly, which is common in sports, it may result in poor quality of life and withdrawal from exercise consequently [4]. Even when there is not an episode of dislocation, the apprehension and loss of confidence in shoulder movements may cause reduced sports activities and a decline in the quality of life [5].

Shoulder instability can be treated non-surgically (mostly in older patients or as a primary treatment option) or surgically (commonly in younger patients and in recurrent cases) [6]. Several surgical procedures have been developed to address shoulder instability [7, 8]. To evaluate the effect of these treatments on symptoms and performance, it is necessary to use an accurate and tested method for measurement. The variables examined during the clinical examination, even when performed by experienced physicians, have been shown to have low reliability and weak correlation with patients' subjective estimation of their performance [9–11]. This lowers their value as a reliable measure for patient functional evaluation.

Patient-reported outcome measures (PROMs) convert the qualitative experiences of patients into quantitative data. Although they are mainly utilized in clinical research, they can assist health professionals in the treatment process and clinical follow-ups, as they provide the capability of measuring the patient's health status, severity and changes in symptoms, and their impact on health and performance from his/her perspective [12]. They also give feedback to the patients about the treatment process and let them monitor their condition which may result in more engagement in achieving the PROMs outcomes [12, 13]. Additionally, PROMs data can be used to determine health policies [14].

In various studies, different PROMs have been used to quantify the assessment of performance and quality of life in patients with shoulder instability [15]. The majority of them assess the general state of health and function like Short Form 12 (SF-12) [16], or anatomically specific instruments such as disabilities of arm, shoulder and hand (DASH) [17] and constant score [18] which assess disabilities of the upper extremity and shoulder dysfunction, respectively. Disease-specific measures are more sensitive for detection and quantifying small changes in patients’ conditions related to specific disorders [19, 20]. Among the limited number of disease-specific PROMs which have been designed to address shoulder instability, the Western Ontario Shoulder Instability Index (WOSI), developed by Kirkley et al. [21], is the most commonly used and recommended instrument [5, 15, 22]. Psychometric properties of the WOSI have been measured in different languages, and the results have shown that WOSI’s validity and reliability are from good to excellent [23–32].

As this PROM has been used in several studies and has been translated into different languages, its availability in Persian language makes it a useful tool for clinic and research. Thus, the present study aimed to translate the WOSI and implement a cross-cultural adaptation of it for the Persian-speaking population, and to determine the measurement properties of the Persian version of the WOSI in patients with shoulder instability in terms of reliability, validity, floor and ceiling effect, measurement error and minimal detectable change.

## Material and methods

### Translation

After obtaining permission from the copyright holder (SG), the translation process was performed according to MAPI Institute instructions [33]. Two independent translators translated the original WOSI (all items including instructions) into Persian (Forward versions A1 and A2). A single translation was obtained after a reconciliation meeting between two translators and the project manager (EK). An official translator reviewed and minimally edited the draft (Forward version B). Forward version B was translated backward into English by another independent translator (Backward translation). The original developer (SG) and the project manager (EK) compared the backward translation with the original WOSI and established Forward version C. Some adaptations were made following a review by four experienced shoulder disease experts (Forward version D). As part of the cognitive debriefing step, five patients completed Forward version D in the presence of the project manager. Interviews were conducted to determine whether the questions were clear and understandable. According to the comments of the patients, a couple of words were replaced (Final version) (Additional file 1). The final edits and adaptations were approved by the developer.

### Patient-reported outcome measures (PROMs)

Meta-analyses have shown that paper-administered PROMs are quantitatively comparable with electronic PROMs (ePROM) [34, 35]. Due to the outbreak of Covid-19, to minimize direct contact, length of stay and the frequency of patients’ visits, we created a web-based version of PROMs.

#### Western Ontario Shoulder Instability Index (WOSI)

A 21-item PROM that was developed as a disease-specific PROM to measure the function and symptoms of patients with shoulder instability during the preceding week [21]. The WOSI contains four domains which include physical symptoms (10 questions), function in sports/recreation/work (4 questions), Lifestyle function (4 questions), and emotions (3 questions). Each item is answered in a range of 0–100 using the Visual Analogue Scale (VAS). Scores of all items are added up to determine a total score between 0 and 2100, with a higher score indicative of worse shoulder function. A web-based version of WOSI was designed using an electronic VAS in the current study. It consisted of a slider that the patients could drag and anchor it to their preferred level from a minimum of 0 to a maximum of 10. The scores were visible for the respondents as they selected them.

#### Oxford shoulder instability score (OSIS)

The OSIS is a disease-specific PROM that measures the function and therapeutic outcomes of patients with shoulder instability [36]. The original version, which was developed by Dawson et al. [36], has excellent internal consistency (Cronbach's alpha = 0.91) and reliability (ICC = 0.97). It consists of 12 five-choice Likert-type questions, with 0 for the best and 4 for the worst. The final score is calculated from the total score of each item, that ranges from 0 to 48 with which 0 is the level of best function with no pain. The Persian version of the OSIS [37] demonstrated excellent reliability (Cronbach's alpha = 0.90 and ICC = 0.94) and good convergent validity, as compared with the VAS and DASH (Pearson correlation coefficient of 0.79 and 0.84, respectively). We used a web-based version of the Persian OSIS.

#### Disabilities of arm, shoulder and hand (DASH)

The DASH is a 30-item PROM that assesses upper limb function over the preceding week. The items are rated on a 5-point Likert-type scale, with 1 representing no dysfunction and 5 representing the highest dysfunction. It includes four subdomains: difficulties in physical function; symptoms of pain, tingling, weakness and stiffness; dysfunction in social activities, work and sleep; and psychological impact [17]. The responses to the DASH items are added to form the raw score. Using the formula: [(raw score/number of responses) − 1] × 25, the DASH scores out of 100 are calculated [38]. Higher scores indicate more disability. The Persian DASH was validated against the functional scales of the Short Form 36 health survey questionnaire (SF-36) with Pearson correlation coefficient ranging from − 0.25 to − 0.72, and the VAS of pain with a correlation of 0.52. It established good test–retest reliability (ICC = 0.82) and excellent internal consistency (Cronbach’s alpha = 0.96) [39]. In the current study, we used a web-based version of the Persian DASH.

#### Oxford shoulder score (OSS)

A 12-item PROM that was designed to evaluate the function of patients with shoulder problems other than instability [40]. It consisted of five-choice Likert-type questions with 4 as no symptom/dysfunction and 0 as the most severe symptoms or dysfunction. The total score is calculated by summing the scores of the items ranging from 0 to 48. The Persian OSS was validated against the SF-36 subdomains with a moderate (physical functioning, role physical, physical component summary) to strong (bodily pain) correlation coefficient. The Pearson correlation coefficient of 0.59 indicated a moderate to strong correlation with the DASH. Cronbach's alpha of the Persian OSS was 0.93 and the ICC was 0.93, indicating high internal consistency and reliability [41]. We used a web-based version of the Persian OSS.

### Patients

According to the recommendations for reliability studies, a minimum of 50 subjects was determined for the study sample size [42, 43]. The study population was recruited from patients referred to a shoulder outpatient clinic and from a private shoulder surgery office, from February 2020 to March 2021.

The clinical diagnosis of shoulder instability was made by history (pain, feeling of instability, or recurrent dislocations) and physical examination, and confirmed with radiographic and MRI studies by an experienced shoulder surgeon (MNA). The inclusion criteria for this study were: clinical diagnosis of shoulder instability and age over 16 years old. The exclusion criteria were: associated fractures (clavicle, scapula, glenoid or proximal humerus), associated acute or extensive rotator cuff injury, degenerative, infectious or inflammatory articular diseases, fixed shoulder dislocations, malignancy, cognitive impairment, and inability to read and write.

**Fig. 1:**
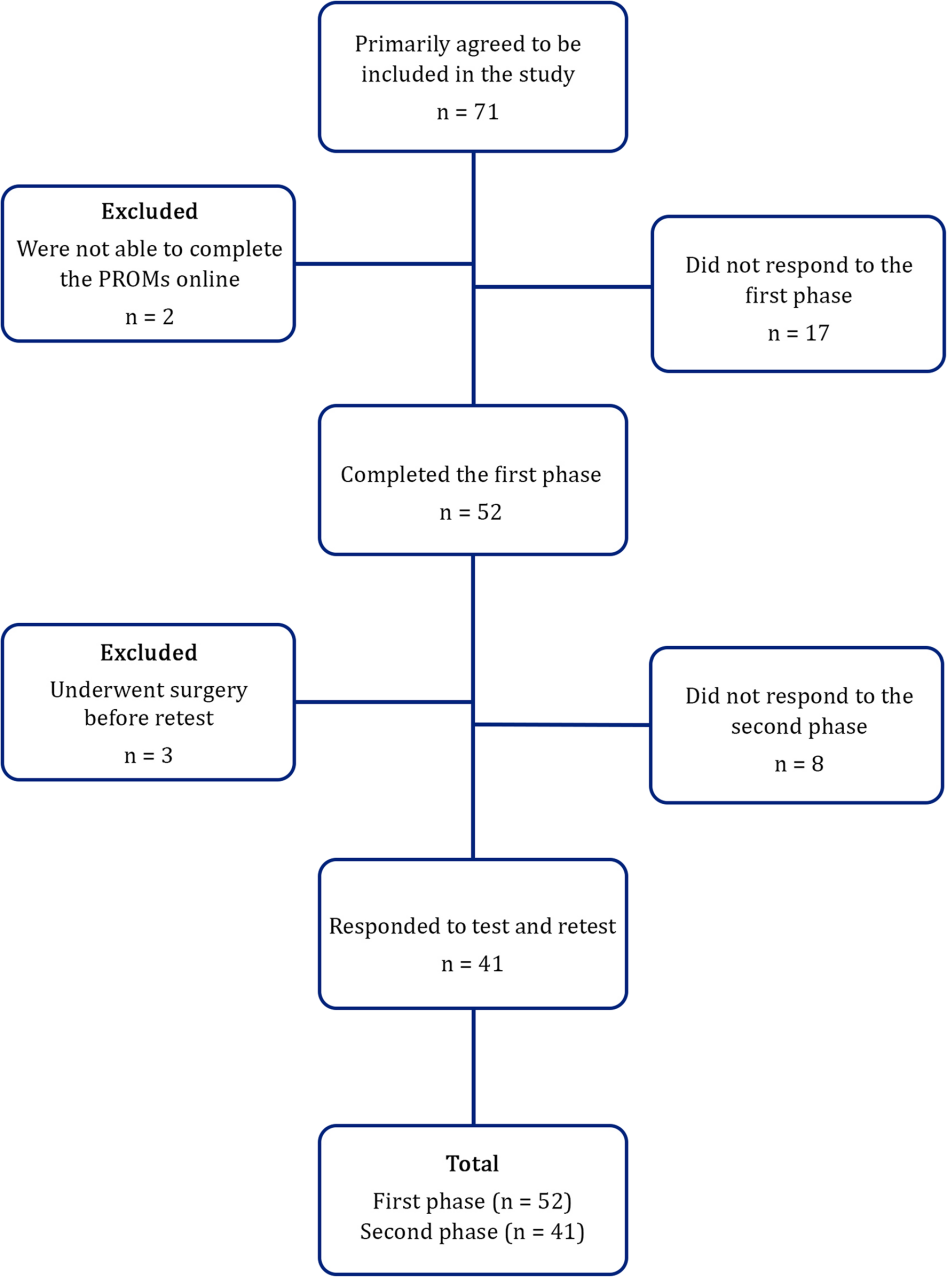
Flowchart of patient inclusion

After the diagnosis of shoulder instability was confirmed, the patient was informed about the project. After his/her verbal consent, a message enclosing a hyperlink was sent to the patient's cellphone. By opening the hyperlink, a web page provided with written information about the study was displayed. By reading the information and accepting the written consent, the patient could start to answer the questions. After a week, another message with another hyperlink to the electronic version of the Persian WOSI was sent to the patient. Between the initial diagnosis (which was at the same time as the first administration) and re-administration of the WOSI, patients were on the waiting list for surgery and did not receive other therapeutic interventions. Patients were contacted if they did not complete the PROMS within a week at each phase (Fig. 1).

### Measurement properties

#### Reliability

Reliability is the ability of an instrument to record the consistent results in a patient with an unchanged condition, during repeated measures [44]. According to the COSMIN consensus, the reliability domain includes three measurement properties: internal consistency, test–retest reliability, and measurement error [44]. Internal consistency is defined as the interrelatedness between items [44]. Cronbach’s alpha coefficient is the most commonly applied index of internal consistency. It ranges between 0 and 1 and is considered strong when approaches 0.90 [45]. Reproducibility or test–retest reliability evaluates the ability of an instrument to maintain consistency over time [44]. The Intraclass correlation coefficient (ICC) is the most widely used index for test–retest reliability of quantitative data [46]. Measurement error has been defined as the systematic and random error of a patient’s score that is not attributed to true changes in the construct to be measured [44]. Minimal detectable change (MDC) is the smallest measurable change that goes beyond the measurement error. Thus, it is safe to conclude that measured changes beyond this value are the result of real changes and not measurement errors [45].

#### Validity

Validity is an index that shows whether an instrument measures what is intended to measure. Content validity of a PROM determines whether it adequately reflects the construct to be measured [44, 45]. Face validity, as an aspect of content validity, evaluates whether the appearance of the designed instrument is consistent with the construct to be measured [44]. The content validity of a PROM is determined by evaluating its relevance, comprehensiveness and comprehensibility with the target population and health professionals [45, 47]. In our study, the final draft was reviewed by two sports medicine specialists and two orthopedic surgeons experienced in shoulder problems. Additionally, five patients were interviewed and their opinions were obtained after completing the WOSI in the presence of one of the co-investigators. Items, response options, and instructions were evaluated by professionals and patients for relevance, comprehensiveness, comprehensibility, and appearance.

Construct validity of an instrument addresses the consistency of the scores to the characteristics it purports to measure [44]. It can be determined by evaluating how well the scores correlate with the gold standard. In the absence of the gold standard, it can be performed by assessing the correlation with other instruments which measure a similar construct (convergent validity) [45].

#### Statistical analysis

The statistical analysis was performed using the IBM SPSS Statistics for Windows, version 23.0 (IBM Corp., Armonk, NY, USA).

For Internal consistency, Cronbach's alpha was calculated. The values outside 0.70 and 0.95 were considered to have low or inappropriately high internal consistency, respectively [44, 45]. ‘Cronbach’s Alpha if Item Deleted’ and ‘Corrected Item-Total Correlation’ evaluate the correlations between each item score and the total score. The desired result was that Corrected Item-Total Correlations stay higher than 0.3 and Cronbach's alpha does not increase after removing any item [48].

For test–retest reliability, the ICC was calculated. The values between 0.75 and 0.9 were considered good and over 0.9 were considered excellent [46]. Two-way mixed-effect model with absolute agreement was used to evaluate the ICC [46].

Measurement error was determined using the standard error of measurement (SEM) by calculating the root of mean square error that was obtained from the ANOVA [45]. As this estimate of the SEM is independent of the ICC, it allows for more consistency in interpreting the values of the SEM [49]. The MDC is based on the SEM, obtained from the formula MDC = 1.96 × √2 × SEM [45].

The use of parametric tests has been demonstrated as a robust method for analyzing summed Likert scale scores even when sample sizes are small, and distributions are non-normal [50–52]. Therefore, the Pearson correlation as a parametric test was considered appropriate for evaluating construct validity. By calculating Pearson correlation coefficient, the scores of the WOSI were compared with the scores of the DASH and the OSS as anatomy-specific PROMs, and OSIS as disease-specific PROM which, similar to WOSI, evaluates the functional limitations in shoulder instability [53]. Additionally, the subdomain scores of the WOSI were compared with the subdomain scores of the DASH. The value of the coefficient varies from − 1 to + 1. A correlation coefficient of 0.4 or less was considered weak, 0.4–0.6 moderate, and above 0.6 was considered strong [54]. The a priori hypothesis for the expected correlations of Persian WOSI is shown in Table 1. Since these PROMs measured symptoms and function of a common anatomic area, we expected high correlations (≥ 0.6) between WOSI and DASH [23], and WOSI and OSS [31]. A higher correlation (≥ 0.7) was expected between WOSI and OSIS as disease-specific PROMs [55]. High correlations (≥ 0.6) were expected in similar subdomains of the WOSI and DASH. The highest correlation (≥ 0.7) was expected between WOSI symptoms and DASH symptoms, as the main measured subdomain of both PROMs [24]. It was expected that these correlation coefficients would be lower (≤ 0.5) between subdomains of the WOSI and DASH that measure non-similar aspects of the construct (WOSI lifestyle/emotions with DASH symptoms/physical function). Construct validity was considered as desirable when 75% of the results agreed with the hypotheses [42].

**Table 1:** The predefined hypotheses for the construct validity of the Persian WOSI

WOSI with DASH: > 0.6
WOSI physical symptoms with DASH physical function: > 0.6
WOSI physical symptoms with DASH symptoms: > 0.7
WOSI sport/recreation/work with DASH physical function: > 0.6
WOSI lifestyle with DASH physical function: < 0.5
WOSI lifestyle with DASH symptoms: < 0.5
WOSI lifestyle with DASH psychosocial: > 0.6
WOSI emotions with DASH physical function: < 0.5
WOSI emotions with DASH symptoms: < 0.5
WOSI emotions with DASH psychosocial: > 0.6
WOSI with OSS: > 0.6
WOSI with OSIS: > 0.7 or > 0.1 higher than that of DASH and OSS

*WOSI* Western Ontario Shoulder Instability Index, *DASH* disabilities of arm, shoulder and hand, *OSS* Oxford shoulder score, *OSIS* Oxford shoulder instability score

## Results

As indicated by the professionals, the Persian WOSI was considered comprehensive and relevant for shoulder instability. To enhance the clarity of the items, professionals recommended that ‘clicking, cracking or snapping’ in item 5 and ‘roughhousing or horsing around’ in item 17 be rephrased with more proper equivalents according to the expressions practically used in the clinic. To prevent distraction, ‘during the last week’ was also suggested to be added to each item. Patients reported that the Persian WOSI was easy to complete, well understood, and addressed their symptoms adequately in a way that they experienced or were preoccupied with. Items that were ambiguous were discussed and word replacements were suggested for items 6 and 21. The final word replacement/addition was approved by the developer (SG).

A total of 52 patients responded to the first phase and 41 patients responded to the second phase. The detailed Patients’ characteristics are shown in Table 2.

**Table 2:** Patients’ characteristics

Patients	69
Responded to the 1st phase	52 (75.3%)
Age (years)	31 ± 8.8
Gender	Male: 38 (73.1%)
	Female: 14 (26.9%)
Work group	Manual: 11 (21.1%)
	Non-manual: 41 (76.9%)
Sport (recreational)	12 (23%)
Etiology of instability	
Traumatic	22 (42.3%)
Atraumatic	30 (57.7%)
Responded to the 2nd phase	41 (83.6%)
Days between test and retest (mean ± SD)	12.6 ± 5.1

*SD* standard deviation

Internal consistency was assessed using data from patients who participated in the first phase. Cronbach's alpha coefficient for total WOSI score was 0.93 (*p* value < 0.01) and ranged from 0.79 to 0.88 for four subdomains (*p* value < 0.01) (Table 3).

**Table 3:** Reliability measures summary

	Test mean score (SD)	Retest mean score (SD)	Cronbach's alpha	ICC [95% CI]	SEM
WOSI total	1370.4 (430.0)	1478.0 (472.5)	0.93	0.90 [0.81, 0.95]	174.5 (8.30%)
Physical symptoms	553.8 (233.6)	634 (249)	0.88	0.84 [0.70, 0.91]	
Sport/recreation/work	293.2 (99.2)	312 (98)	0.80	0.82 [0.67, 0.90]	
Lifestyle	289.0 (98.5)	282 (106)	0.79	0.91 [0.83, 0.95]	
Emotions	245.5 (61.7)	249 (69)	0.79	0.89 [0.79, 0.94]	

*SD* standard deviation, *ICC* intraclass correlation coefficient, *CI* confidence interval, *SEM* standard error of measurement

Alpha stayed consistent with all items and ‘Corrected item-total correlation’ coefficients ranged from 0.42 to 0.82 (Table 4).

**Table 4:** Total-item statistics

WOSI questions	Corrected item-total correlation	Cronbach's alpha if item deleted
Item 1	0.65	0.92
Item 2	0.60	0.92
Item 3	0.63	0.92
Item 4	0.64	0.92
Item 5	0.55	0.92
Item 6	0.59	0.92
Item 7	0.51	0.92
Item 8	0.63	0.92
Item 9	0.52	0.92
Item 10	0.70	0.92
Item 11	0.52	0.92
Item 12	0.82	0.92
Item 13	0.59	0.92
Item 14	0.62	0.92
Item 15	0.51	0.92
Item 16	0.68	0.92
Item 17	0.62	0.92
Item 18	0.59	0.92
Item 19	0.63	0.92
Item 20	0.61	0.92
Item 21	0.42	0.93

*WOSI* Western Ontario Shoulder Instability Index

Test–retest reliability was good to excellent with an ICC of 0.90 (95% CI [0.81, 0.95]). The ICC value for each domain was in the range of 0.79–0.91, which also indicated a good to excellent test–retest reliability of each domain. Using the root of mean square error, the standard error of measurement (SEM) was calculated as 174.5 (8.30%). According to the above-mentioned formula, the MDC value of the Persian WOSI was 484 in the raw score (23.04% of the total WOSI score).

Considering the threshold of 15%, no ceiling or floor effect was observed on the total score and score of the subdomains. Regarding MDC, 2% of the samples were within the minimum range (0–484) and 34.6% within the maximum range (1616–2100).

The correlation between the scores of the WOSI and the OSIS, the OSS and the DASH, and also between the subdomain scores of the WOSI and the DASH was analyzed to assess the construct validity. For all three PROMs, the Pearson correlation coefficient was in the range of 0.6–0.8, which indicated a satisfactory construct validity of the Persian WOSI. The correlation between the subdomains is presented in Table 5. The a priori hypotheses were confirmed in 83.3% (10 out of 12) of correlation evaluations.

**Table 5:** Pearson correlation coefficient

	OSIS	OSS	DASH			
			Total	Physical functions	Symptoms	Psychosocial
WOSI total	0.643^†^	0.759*	0.746*			
WOSI physical symptoms				0.638*	0.725*	0.570
WOSI sport/recreation/work				0.654*	0.531	0.637
WOSI lifestyle				0.576*	0.451*	0.570^†^
WOSI emotions				0.480*	0.495*	0.606*

*WOSI* Western Ontario Shoulder Instability Index, *OSIS* Oxford shoulder instability score, *OSS* Oxford shoulder score, *DASH* disabilities of arm, shoulder and hand

*Hypothesis confirmed

^†^Hypothesis not confirmed

## Discussion

The present study successfully translated and cross-culturally adapted WOSI for Persian speakers. The Persian WOSI demonstrated excellent reliability and validity with no floor and ceiling effect. Similarly, the WOSI was previously translated and culturally adapted in several languages and obtained favorable psychometric properties comparable to those established for the original version [23–32].

The PROM was translated using a standard method. Some of the items needed to be rephrased, without resulting in significant changes in the structure of the questions. Also, according to ‘Corrected Item-Total Correlation’ and ‘Cronbach’s Alpha if Item Deleted,’ there was no need for item deletion. Patients had no difficulty understanding the items/questions and instructions, and the back translation was well comparable with the English version.

Measurement of inter-items reliability by calculating Cronbach's alpha of 0.93 showed strong internal consistency among all questions. The Italian and German translations of WOSI reported similar values of 0.93 and 0.92 [24, 25], respectively. This value has been reported to be 0.95 in the Swedish and Dutch translations [27, 28] and 0.84 in the Japanese translation [32]. All these results are categorized as good to excellent. However, values higher than 0.95 are not desirable and are more indicative of redundancy [56]. Cronbach's alpha was also calculated separately for the four subdomains which ranged from 0.79 to 0.88. This indicated good consistency and interrelatedness of items of each subdomain as well. The Turkish [23], German [25], Arabic [26], and Swedish [27] translations reported similar values ranging from 0.77 to 0.90 for most subdomains. However, a low Cronbach's alpha was reported for the sport/recreation/work subdomain in the Arabic translation (0.56) and the lifestyle subdomain in the Swedish and German translations (0.56 and 0.68, respectively). The two Dutch translations reported these values for the subdomains in a higher range (0.88–0.95) indicating stronger internal consistency.

The test–retest reliability of the Persian WOSI showed good to excellent reliability in repeated measures with an ICC of 0.90. Compared to the ICC reported by Kirkley et al.'s original study (ICC = 0.949) [21], our ICC was lower but still within the range of good to excellent. Some other studies have reported a similar result, with ICC at 0.91 or 0.92 [25, 28, 29, 32]. For the subdomains, the ICC ranged from 0.82 to 0.91, which remained in the range of good (over 0.75) to excellent (over 0.90) and comparable to the results of the original version of WOSI, ranging from 0.719 to 0.941 [21]. These results also agreed with those reported by Salomonsson et al. (0.85–0.91) [27], Hofstaetter et al. (0.87–0.93) [25], van der Linde et al. (0.88–0.90) [28], and Perrin et al. (0.80–0.94) [30] for the subdomains.

The retest interval, sample size and heterogeneity of the samples, optimal administration of the PROMs during retest studies, retest data reassessment, and collecting follow-up data after retest are important factors that affect reliability and needed to be considered when designing a retest study [57]. Although a certain recommended time interval does not exist for retesting, an interval of 7–14 days is generally suggested for health studies [57–59]. Shoulder instability is a result of trauma, overuse microtrauma or ligament laxity which can be managed operatively or non-operatively [60]. The non-operative management consisted of strengthening exercises for rotator cuff and scapular stabilizing muscles for the most part [61, 62]. Thus, there is a little chance of worsening symptoms (in the absence of surgery or an acute new problem, as mentioned in our exclusion criteria) or improving symptoms with strengthening exercises within a week or two. Although achieving a satisfying sample size may not be viable when the interval between test and retest becomes longer, by administrating the retest after 7 days, the optimal level of reliability can be achieved. Therefore, it can be concluded that the ICC obtained by re-administration of the WOSI after an average of 12.6 days in our study, is a product of a more methodologically reliable approach. In studies in which the patients were retested after about 2 weeks [28, 29, 32], the ICC was similar to our study (0.91–0.92).

The SEM and MDC of the Persian version of WOSI were 8.3% and 23.04%, respectively. It indicates that a change of more than 484 scores between two measurements could be considered significant regardless of measurement error. A lower SEM and MDC have been observed in some previous studies (Table 6). As the SEM is related to the ICC value [SEM = SD × √ (1 − ICC)], a higher ICC value due to a shorter interval between test and retest may be a cofactor of this difference. In the study by van der Linde et al. [28] which the retest was administered under similar conditions to our study, similar values for SEM and MDC were reported.

**Table 6:** List of Studies that calculated SEM and MDC for WOSI

	Test–retest interval (days)	SEM (%)	MDC	ICC
Cacchio et al. [24]	3	3.38	9.33	0.95
Ismail et al. [26]	3–7	4.29	11.9	0.96
Perrin et al. [30]	7	5.7	15.9	0.93
van der Linde et al. [28]	13	8.3	23	0.92
Our study	12.6	8.30	23.04	0.90

*SEM* standard error of management, *MDC* minimal detectable change, *WOSI* Western Ontario Shoulder Instability Index, *ICC* intraclass correlation coefficient

In the present study, no patients scored neither the minimum nor the maximum score (0 and 2100, respectively). The distribution of individuals' scores indicated that, by definition, the instrument had no ceiling or floor effect. The total score of 2% of the respondents was in the MDC range from the minimum score and 34.6% of them was in the MDC range from the maximum score. This was expected since the respondents were all patients with shoulder instability and not a healthy population. From a clinical point of view, given that a third of our patients was in the MDC range from the maximum score, tracking their progress would be associated with a higher possibility of measurement error, if their conditions become worse.

The findings of the present study suggested satisfactory construct validity, as shown by high correlation of WOSI with DASH, OSS, and OSIS. The Pearson correlation coefficient value showed a significant relationship for the DASH (0.746) which was in agreement with the original validation of the WOSI (0.76) [21] and Italian version (0.79) [24]. Basar et al. reported a slightly lower but still significant correlation with DASH (0.67) [23]. A high correlation was also observed for OSS (0.759) and similarly in the Danish version (0.79) [31]. Although the correlation coefficient of OSIS, as an instrument that measures the same construct, was not higher (0.643) than that of OSS and DASH, it was within the range that considered as a strong correlation (over 0.6). The correlation of WOSI with OSS, DASH, and OSIS was also studied in an adaptation study of Dutch WOSI, and they were within a narrow range (0.79, 0.81 and 0.82 respectively) [28]. Collectively, these data indicate that although the WOSI and OSIS examine functional limitations due to shoulder instability, the content of the items may have overlap with other shoulder and upper extremity disorders.

One of the weaknesses of our study was that we were not able to recruit bigger sample size due to the limited number of patients referred to the orthopedic outpatient clinics during the COVID-19 pandemic. Thus, with 41 patients participating in the retest phase, we could not reach the recommended minimum of 50 patients for the retest phase [42].

In order to avoid multiple visits and reduce the patient’s length of stay at the clinic, we designed an electronic version of the PROMs. Previously, Eshoj et al. validated the electronic version of the Danish WOSI in comparison with its paper-based version [31]. According to the ISPOR (International Society for Pharmacoeconomics and Outcomes Research) taskforce, by migrating from the paper-based PROMs to the electronic version, equivalence studies are not required if the changes are minor [63]. However, the lack of an equivalence study for the Persian WOSI can be considered a limitation. The use of this method caused some patients to be excluded from the study, as they were internet novices and not familiar with online questionnaires. This method, however, made it possible for patients to answer questions at home and allowed for no questions to remain unanswered.

## Conclusion

The results of the present study indicate that the Persian adaptation of the WOSI is a valid and reliable self-administered PROM. It can be administered via the internet and completed easily by Persian-speaking patients with shoulder instability.

## Supplementary Information

**Additional file 1:** The Persian WOSI.

## Data Availability

The dataset supporting the conclusions of this article is accessible in "related files".
