# Supplementary material for: Cross-cultural adaptation, validity and reliability of the Persian translation of the Western Ontario Shoulder Instability Index (WOSI)
Source: J Orthop Surg Res. 2023 Mar 7;18:174. doi: 10.1186/s13018-023-03593-z (PMC9990569; doi:10.1186/s13018-023-03593-z)
Supplement: Supplementary file 1 — Additional file 1: The Persian WOSI. [file 13018_2023_3593_MOESM1_ESM.pdf]

پرسشنامۀ ناپایداری شانۀ وسترن انتاریو

Western Ontario Shoulder Instability Index (WOSI)

قسمت الف: علایم جسمانی

سوالات زیر به علائم جسمانی‌ای می‌پردازد که بخاطر مشکل شانه‌تان تجربه کرده‌اید. در تمام این سوالات، لطفا میزان علایمی که در طی هفتۀ گذشته تجربه کرده‌اید را (با علامت ضربدر(×)، روی خط افقی مربوطه) علامت بزنید.

۱. در طول هفتۀ گذشته، حین انجام فعالیت های بالای سر، چه میزان درد در شانۀ خود تجربه کرده‌اید؟

|-----------,-----------,-----------,-----------,-----------,-----------,-----------,-----------,-----------,-----------|

درد شدید — **هیچ دردی**

۲. در طول هفتۀ گذشته، چه مقدار درد تیرکشنده یا مبهم و ممتد در شانۀ خود تجربه کرده‌اید؟

|-----------,-----------,-----------,-----------,-----------,-----------,-----------,-----------,-----------,-----------|

درد مبهم یا تیرکشندۀ شدید — **هیچ درد مبهم یا تیرکشنده‌ای**

۳. در طول هفتۀ گذشته، چه مقدار ضعف یا کاهش قدرت در شانۀ خود تجربه کرده‌اید؟

|-----------,-----------,-----------,-----------,-----------,-----------,-----------,-----------,-----------,-----------|

ضعف شدید — **هیچ ضعفی**

۴. در طول هفتۀ گذشته، چه مقدار احساس خستگی یا ضعف رفتن در شانۀ خود تجربه کرده‌اید؟

|-----------,-----------,-----------,-----------,-----------,-----------,-----------,-----------,-----------,-----------|

خستگی شدید — **هیچ‌گونه خستگی**

۵. در طول هفتۀ گذشته، چه مقدار صداهای اضافه (صدای تق تق، خش خش، ترق تروق) در شانۀ خود تجربه کرده‌اید؟

|-----------,-----------,-----------,-----------,-----------,-----------,-----------,-----------,-----------,-----------|

بسیار زیاد — **هیچ صدایی**

۶. در طول هفتهٔ گذشته، چه مقدار احساس خشکی در شانهٔ خود داشته‌اید؟

|----------|----------|----------|----------|----------|----------|----------|----------|----------|----------|

خشکی بسیار زیاد — **هیچ‌گونه خشکی**

۷. در طول هفتهٔ گذشته، به‌خاطر مشکل شانه‌تان چه مقدار احساس ناراحتی در عضلات گردن داشته‌اید؟

|----------|----------|----------|----------|----------|----------|----------|----------|----------|----------|

ناراحتی بسیار زیاد — **هیچ‌گونه ناراحتی**

۸ . در طول هفتهٔ گذشته، چه مقدار احساس بی‌ثباتی یا لق بودن در شانهٔ خود داشته‌اید؟

|----------|----------|----------|----------|----------|----------|----------|----------|----------|----------|

بی‌ثباتی بسیار زیاد — **هیچ‌گونه بی‌ثباتی**

۹. در طول هفتهٔ گذشته، چه مقدار نیاز داشته‌اید مشکل شانه‌تان را با به‌کارگیری سایر عضلاتتان جبران کنید؟

|----------|----------|----------|----------|----------|----------|----------|----------|----------|----------|

بسیار زیاد — **هیچ**

۱۰. در طول هفتهٔ گذشته، چه مقدار محدودیت در حرکت شانه‌تان داشته‌اید؟

|----------|----------|----------|----------|----------|----------|----------|----------|----------|----------|

بسیار زیاد — **هیچ**

قسمت ب: ورزش/تفریح/کار

سوالات زیر به این موضوع می‌پردازدکه مشکل شانه‌تان چقدرکار، ورزش یا فعالیتهای تفریحی شما در طی هفتهٔ گذشته را تحت تاثیر قرار داده است. برای هر سوال، لطفا میزان این تاثیر را (با علامت ضربدر(×)، روی خط افقی مربوطه) علامت بزنید.

۱۱. در طول هفتهٔ گذشته، مشکل شانهٔ شما چه مقدار فعالیت‌های ورزشی و تفریحی‌تان را محدود کرده است؟

|----------|----------|----------|----------|----------|----------|----------|----------|----------|----------|

بسیار زیاد — **هیچ**

۱۲. در طول هفتهٔ گذشته، مشکل شانه شما چه مقدار بر توانایی به‌کاربردن مهارت‌های خاص مورد نیاز برای انجام ورزش یا کارتان تاثیر گذاشته است؟ (اگر شانه شما هم بر ورزش و هم بر کار شما تاثیر گذاشته، قسمتی را در نظر بگیرید که بیشترین اثر را گذاشته است.)

|-----------,-----------,-----------,-----------,------------,-----------,-----------,-----------,-----------,-----------|

تاثیر بسیار زیاد ... **بدون تاثیر**

۱۳. در طول هفتهٔ گذشته، چه مقدار احساس کرده‌اید که نیاز دارید در طی فعالیت‌های روزمره از بازوی خود محافظت کنید؟

|-----------,-----------,-----------,-----------,------------,-----------,-----------,-----------,-----------,-----------|

بسیار زیاد ... **هیچ**

۱۴. در طول هفتهٔ گذشته، برای بالا بردن اجسام سنگین تا ارتفاع شانه، چه مقدار مشکل داشته‌اید؟

|-----------,-----------,-----------,-----------,------------,-----------,-----------,-----------,-----------,-----------|

مشکل بسیار ... **هیچ مشکلی**

قسمت پ: سبک زندگی

سوالات زیر به این موضوع می‌پردازد که مشکل شانه‌تان، چقدر سبک زندگی شما را تحت تاثیر قرار داده یا تغییر داده است. مجدداً برای هر سوال، لطفا میزان این تاثیر را در طی هفتهٔ گذشته (با علامت ضربدر(×)، روی خط افقی مربوطه) علامت بزنید.

۱۵. در طول هفتهٔ گذشته، چقدر می‌ترسید که روی شانه‌تان زمین بخورید؟

|-----------,-----------,-----------,-----------,------------,-----------,-----------,-----------,-----------,-----------|

خیلی زیاد ... **اصلا**

۱۶. در طول هفتهٔ گذشته، حفظ آمادگی جسمانی دلخواهتان چقدر سخت بوده است؟

|-----------,-----------,-----------,-----------,------------,-----------,-----------,-----------,-----------,-----------|

بسیار سخت ... **بدون سختی**

۱۷. در طول هفتهٔ گذشته، چقدر حین بازی و شوخی و جست‌وخیز با دوستان یا اعضای خانواده مشکل داشته‌اید؟

|-----------,-----------,-----------,-----------,------------,-----------,-----------,-----------,-----------,-----------|

بسیار زیاد ... **هیچ**

۱۸. بخاطر مشکل شانه‌تان، در طول هفتهٔ گذشته چقدر هنگام خوابیدن مشکل داشته‌اید؟

|-----------|-----------|-----------|-----------|-----------|------------|-----------|-----------|-----------|-----------|

مشکل بسیار زیاد   **هیچ مشکلی**

قسمت ت: احساسات

سوالات زیر به این موضوع می‌پردازد که در طی هفتهٔ گذشته چه حسی در ارتباط با مشکل شانهٔ خود داشته اید. لطفاً برای هر سوال، پاسخ خود را (با علامت ضربدر(×)، روی خط افقی مربوطه) علامت بزنید.

۱۹. در طول هفتهٔ گذشته، چقدر توجه‌تان معطوف به شانه‌تان بوده است؟

|-----------|-----------|-----------|-----------|-----------|------------|-----------|-----------|-----------|-----------|

بسیار زیاد   **به هیچ وجه**

۲۰. در طول هفتهٔ گذشته، چقدر نگران بوده‌اید که مشکل شانهٔ شما بدتر شود؟

|-----------|-----------|-----------|-----------|-----------|------------|-----------|-----------|-----------|-----------|

بسیار زیاد   **به هیچ وجه**

۲۱. در طول هفتهٔ گذشته، بخاطر مشکل شانه‌تان چقدر احساس سرخوردگی و کلافگی داشته‌اید؟

|-----------|-----------|-----------|-----------|-----------|------------|-----------|-----------|-----------|-----------|

بسیار زیاد   **به هیچ وجه**
